# The effectiveness of CBT-based NLP-enabled AI conversational agents for mental health intervention: a systematic review and meta-analysis

**DOI:** 10.1038/s41746-026-02886-x

**Published:** 2026-06-12

**Authors:** Yaming Hang, Wenzhi Wu, Yi Feng, Kai Yan, Yinuo Liu, Xiyao Xiao, Zhihong Qiao

**Affiliations:** 1 Fujian Province Key Laboratory of Applied Cognition and Personality, Zhangzhou, China; 2 Institute of Applied Psychology, Minnan Normal University, Zhangzhou, China; 3 School of Education and Psychology, Minnan Normal University, Zhangzhou, China; 4 National Demonstration Center for Experimental Psychology Education, Faculty of Psychology, Beijing Normal University, Beijing, China; 5 Mental Health Center, Central University of Finance and Economics, Beijing, China; 6 School of Sociology and Psychology, Central University of Finance and Economics, Beijing, China; 7 Department of Psychology, Renmin University of China, Beijing, China; 8 Lingxin AI, Beijing, China

**Keywords:** Diseases, Health care, Psychology, Psychology

## Abstract

Natural language processing (NLP)-enabled artificial intelligence (AI) conversational agents (CAs) are increasingly adopted in digital mental health interventions, yet the efficacy of such CAs grounded in cognitive behavioral therapy (CBT) remains unclear. This study aims to examine the intervention effectiveness of CBT-based NLP-enabled AI CAs in various mental health problems. A total of 15 randomized controlled trials with 1737 participants were included in the analysis. The results indicated that CBT-based NLP-enabled AI CAs showed a small to moderate effect on depressive symptoms and a small effect on negative affect; while the effects on generalized anxiety, stress, and positive affect were not significant after adjusting for publication bias. Subgroup analyses provided preliminary evidence that multi-modal CAs may be more effective than single-modality CAs in reducing depressive symptoms, and that the absence of psychoeducational content was associated with larger post-test effect sizes. Notably, meta-regression revealed that higher-quality studies reported larger effect sizes, suggesting that the true efficacy of these interventions may be underestimated in the current literature. In addition, younger age was associated with a greater reduction in depressive symptoms. These findings underscored the potential of CBT-based NLP-enabled AI CAs in addressing certain mental health issues and in certain populations.

## Introduction

Mental health issues represent a critical global public health challenge, which significantly affects the lives of millions of people worldwide, leading to profound consequences for both communities and economies^1^. The global burden of these disorders has been significantly exacerbated by the COVID-19 pandemic, which led to decreased mental well-being and a notable increase in the prevalence of mental health issues, particularly depression and anxiety^2^. These challenges underscore the urgent need for effective and accessible interventions.

Cognitive Behavioral Therapy (CBT) is widely recognized as one of the most effective psychological interventions for a range of mental disorders, including depression. Meta-analytic evidence indicates that CBT produces moderate to large effects for depressive symptoms (*g* = 0.79; 95% CI [0.70, 0.89])^3^. CBT encompasses a range of cognitive, behavioral, and emotion-focused techniques aimed at reducing symptoms, improving functioning, and alleviating psychological distress. Commonly used techniques include cognitive restructuring, behavioral activation, and exposure-based strategies^4^. Despite its demonstrated effectiveness, the traditional mode of CBT delivery—typically involving face-to-face sessions with a therapist—encounters several practical barriers, including the pervasive stigma associated with seeking mental health care, limited availability of qualified therapists, and significant financial pressures on healthcare systems^5^. In addition, the implementation of CBT in community settings faces substantial challenges. Research highlights multiple barriers, including limited training, supervision, time constraints, and difficulty adapting treatments to individual needs or comorbidities^6^. Studies in community mental health settings reveal that fewer than one-quarter of patients diagnosed with anxiety disorders receive CBT^7^, while access to evidence-based psychological treatments continues to decline^8^. Additionally, therapists’ self-reported theoretical orientation does not reliably predict CBT competency^9^, and even trained clinicians may drift from the model over time, reducing treatment fidelity^10^. Furthermore, the financial costs of conventional CBT intervention, particularly in cases of treatment resistance, remain substantial^11^.

Conventional digital mental health interventions offer a promising solution to these challenges^12^. These approaches deliver evidence-based psychotherapies through digital platforms, including email or text-based support, video counseling, and self-help mobile applications^13^. Previous studies have demonstrated the therapeutic effectiveness of digital therapies, such as Computer-Based Cognitive Behavioral Therapy (CCBT) and Internet-Based Cognitive Behavioral Therapy (ICBT) for depression (*g* = 0.80; 95% CI [0.20, 1.40]) and anxiety (*g* = 0.21; 95% CI [0.09, 0.33]), which deliver CBT interventions through software and online platforms^14–16^.

Although digital forms of CBT have helped alleviate some issues related to stigma, shortage of mental health professionals and accessibility to some degree^17^, they still face several challenges, such as limited interactivity and relatively high dropout rates^18^. The structured nature of self-guided courses and exercises does not allow for real-time responses tailored to the user’s current state, relying heavily on the user’s personal motivation to persist. This shortcoming can hinder participant engagement and retention, particularly over the long term^19^. These challenges underscore the critical need for more flexible and adaptive CBT models that prioritize enhanced interactivity and personalized user experiences, ultimately catering more effectively to the diverse requirements of different populations.

Conversational Agent Interventions represent a new wave of digital mental health interventions designed to address these challenges^20^. A key feature of Conversational Agents (CAs) in mental health is their interactivity, which allows users to engage in a conversational process. This interactivity can complement traditional digital interventions, which may also deliver CBT skills such as cognitive restructuring or behavioral activation, but in a less interactive, more didactic format^21^. Recent integrative reviews suggest that artificial intelligence (AI)-driven psychological interventions, particularly CBT-oriented CAs, represent a promising evolution beyond conventional digital therapies^22,23^. While these CAs showed inconsistent performance in treating various mental health conditions, they demonstrate promising results in reducing depression and anxiety symptoms. Unlike traditional digital CBT tools, CAs provide real-time interactions and feedback, thereby fostering greater user engagement and adherence to therapeutic protocols. By simulating human-like dialogues, CAs deliver a more dynamic and responsive therapeutic experience. However, a large proportion of current CAs operate on rule-based systems, which are based on recognizing the lexical form of the text with pre-set answers^24^. While rule-based CAs can be effective to some extent, they are limited in their ability to fully comprehend user context and intention. Advances in AI, particularly in natural language processing (NLP) and machine learning, have facilitated the development of more advanced AI-driven CAs, including retrieval-based and generative CAs. These CAs employ AI techniques (e.g., NLP or machine learning) to guide the flow and content of the interaction. Unlike traditional systems lacking NLP capabilities, these agents are able to interpret user intent, analyze conversational context, and retrieve or generate contextually appropriate responses based on users’ inputs^25^. Retrieval-based CAs leverage learned representations and similarity matching to select the most contextually relevant responses from an evolving response set, enabling improved adaptation to users’ inputs over time. In contrast, generative CAs employ deep learning-based language models to produce novel responses dynamically, allowing for greater conversational flexibility and personalization. Together, these AI-driven approaches enable CAs to process more complex information and provide increasingly adaptive, personalized, and sophisticated responses to mental health needs^26^. At present, most NLP-enabled AI CAs in the mental health field are based on retrieval-oriented architectures, while generative models remain at an early stage of development with relatively limited empirical support. Thus, the term “NLP-enabled AI CAs” is therefore used to refer mainly to retrieval-based approaches, while acknowledging the emerging role and distinct evidence base of generative CAs.

Despite the promising results from previous meta-analyses on the effectiveness of CAs in addressing various mental health issues, these studies often include a wide range of CA types^20,27^. For example, existing reviews have not sufficiently differentiated between the efficacy of CAs that follow predefined scripts and those that utilize advanced AI techniques like NLP and machine learning to deliver more personalized and adaptive interventions^28^. Additionally, all of the previous meta-analyses examined the efficacy of CAs based on diverse therapeutic approaches^24^. This heterogeneity introduces significant variability in outcomes, making it difficult to isolate the specific effects of CBT-based NLP-enabled AI CAs. The present study aims to provide a more focused and differentiated synthesis by specifically examining CBT-based NLP-enabled AI CAs. By narrowing the scope to a more theoretically and technologically coherent subgroup of interventions, this review seeks to clarify their efficacy across different mental health outcomes, explore potential moderators of treatment effects, and provide more precise clinical and methodological insights for the refinement of CBT-based AI interventions.

## Results

### Search results

**Fig. 1:**
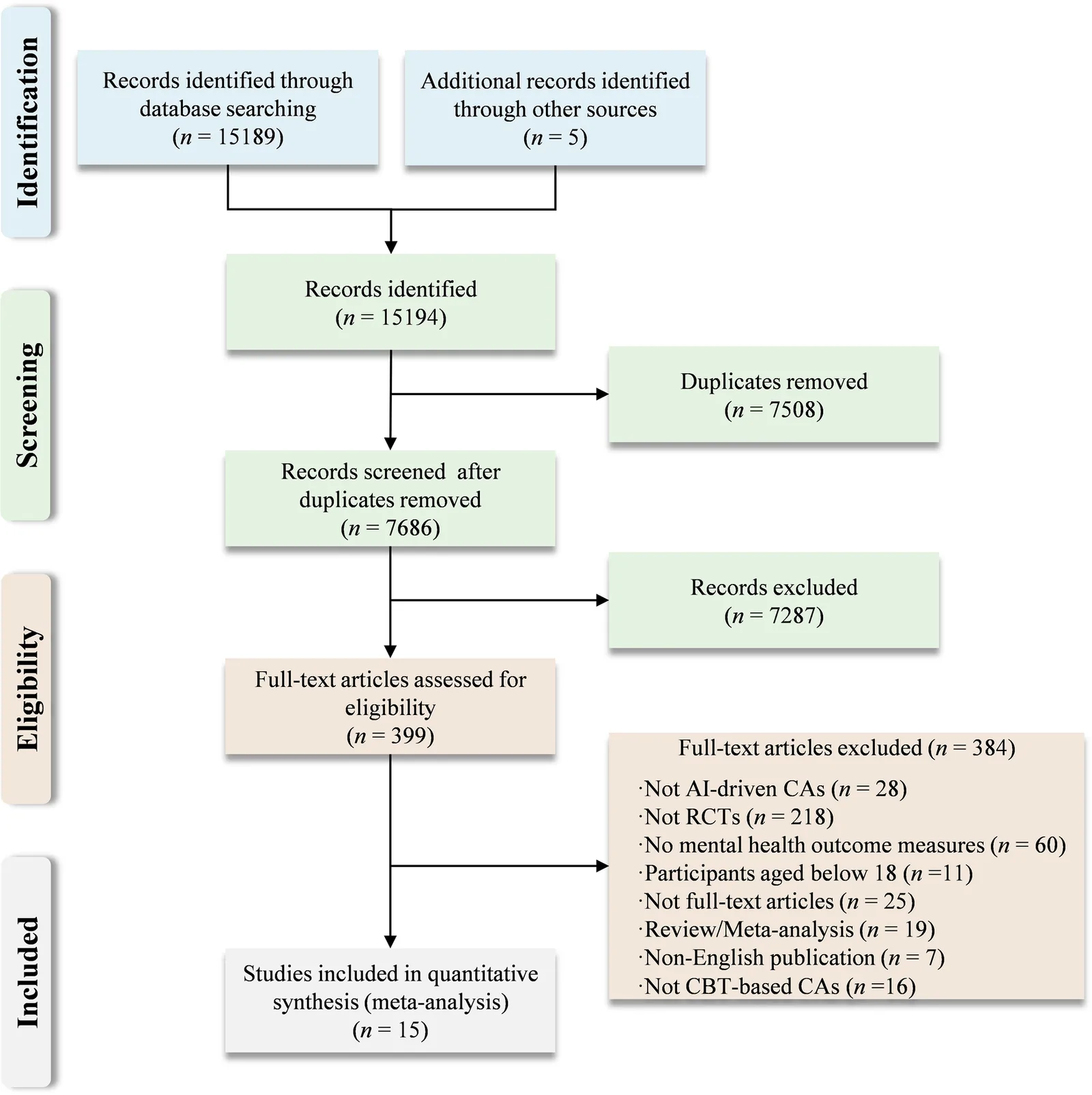
Flowchart of the study inclusion procedure. This figure describes the process of selecting and screening studies, from database searching to studies that met full eligibility criteria. This figure was generated by R (version 4.5.1) https://www.R-project.org/^71^.

A total of 15,189 potential articles were initially identified, with 5 additional papers found through hand-searching reference lists. After removing 7508 duplicates, 7686 unique papers remained. The titles and abstracts were then screened, leading to the exclusion of 7287 records. The remaining 399 records were identified as potentially eligible, and their full texts were reviewed. The study inclusion process followed the PRISMA guidelines^29^ (see Fig. 1). This procedure resulted in the inclusion of 15 published articles in the systematic review and meta-analysis.

### Study characteristics

Sample sizes of the 15 included studies ranged from 27 to 457, and the total number of participants across all studies was 1737 (see Table 1). This meta-analysis included studies conducted on clinical (*n* = 3), subclinical (*n* = 8), and nonclinical (*n* = 4) samples. Thirteen of the 15 studies utilized retrieval-based CAs, one study employed generative CA, and one study used both retrieval-based and generative CAs. The interaction modes included text-based (*n* = 12), voice-based (*n* = 1), and multimodal (*n* = 2) CAs. Eleven studies used CAs based on CBT, while four studies employed CAs based on integrative approaches incorporating CBT. The duration of interventions ranged from 2 to 16 weeks. Regarding CBT skills included in CAs, 13 of the 15 studies reported the inclusion of cognitive restructuring, and 5 of the 15 studies reported the inclusion of behavioral activation. However, only one study explicitly reported the inclusion of exposure training^30^. The detailed information about commonly included CBT skills in NLP-enabled AI CAs can be seen in Supplementary Table 2.

**Table 1:** Study characteristics

First author, year (country)	Sample type	*N*	%—total Female	Mean age	CA name	Delivery platform	Response generation approach	Interaction mode	Therapeutic orientation	CBT skills included	Intervention length (weeks)	Control	Outcome measures
Burton et al. 2016^58^ (ROU, ES, GB)	Clinical	27	67.0	38.50	Help4 Mood	Standalone app	Retrieval based	Text-based	CBT only	Mood tracking, Cognitive restructuring, Behavioral activation, Relaxation exercises	4	Active control	BDI-2, QIDS-SR, DAS-SF, EQ-5D-5L
Danieli et al. 2022^59^ (ITA)	Subclinical	45	78.0	55.58	TEO	Standalone app	Retrieval based	Text-based	CBT only	ABC (Activation, Belief, and Consequence) technique, Relaxation exercises	8	Waitlist or assessment only	PHQ-8, GAD-7, PSS
Fitzpatrick et al. 2017^60^ (US)	Subclinical	70	67.0	22.20	Woebot	Standalone app	Retrieval based	Text-based	CBT only	Psychoeducation, Mood tracking, Cognitive restructuring, Behavioral Activation, Mindfulness	2	Information only	PHQ-9, GAD-7, PANAS
Fulmer et al. 2018^61^ (US)	Subclinical	75	69.0	22.90	Tess	Instant messenger platform	Retrieval based	Text-based	Integrative	Mood tracking, Relaxation exercises, Cognitive restructuring, Mindfulness	2–4	Information only	GAD-7, PHQ-9
He et al. 2022^40^ (CN)	Subclinical	125	80.0	18.78	XiaoE	Instant messenger platform	Both	Multimodal	CBT only	Thoughts identification, Cognitive restructuring, Relaxation exercises, Mindfulness	1	Active control	PHQ-9
Jang et al. 2021^62^ (KOR)	Subclinical	46	57.0	25.10	Todaki	Standalone app	Retrieval based	Text-based	CBT only	Psychoeducation, Relaxation exercises, Mindfulness	4	Information only	QIDS-SR, SAS, PSS
Karkosz et al. 2024^63^ (POL)	Subclinical	68	72.0	25.68	Fido	Instant messenger platform	Retrieval based	Text-based	CBT only	Psychoeducation, Cognitive restructuring/Socratic questioning, ABC technique	2	Information only	CESD-R, PHQ-9, PSWQ, STAI, PANAS, SWLS, R-UCLA
Klos et al. 2021^64^ (ARG)	Nonclinical	73	87.0	NR	Tess	Instant messenger platform	Retrieval based	Text-based	Integrative	Mood tracking, Relaxation exercises, Cognitive restructuring, Distress tolerance, Mindfulness	8	Information only	PHQ-9, GAD-7
Liu et al. 2022^65^ (CN)	Subclinical	83	55.0	23.08	XiaoNan	Instant messenger platform	Retrieval based	Multimodal	CBT only	Mood tracking, Cognitive restructuring	16	Active control	PHQ-9, GAD-7, PANAS
MacNeill et al. 2024^66^ (CAN)	Clinical	76	69.0	42.87	Wysa	Standalone app	Retrieval based	Text-based	Integrative	Mood tracking, Cognitive restructuring, Mindfulness, Contingency management	4	Waitlist or assessment only	PHQ-9, GAD-7, PSS
Oh et al. 2020^30^ (KOR)	Clinical	41	51.0	40.53	Todaki	Standalone app	Retrieval based	Voice-based	CBT only	Mood tracking, Cognitive restructuring, Exposure training, Relaxation exercises	4	Information only	HADS
Prochaska et al. 2021^67^ (US)	Subclinical	152	65.0	40.00	Woebot	Standalone app	Retrieval based	Text-based	CBT only	Psychoeducation, Mood tracking, Cognitive restructuring, Behavioral Activation, Mindfulness	8	Waitlist or assessment only	PHQ-8, GAD-7
Sabour et al. 2023^68^ (CN)	Nonclinical	247	80.0	30.90	Emohaa	Instant messenger platform	Generative	Text-based	CBT only	Mood tracking, Cognitive restructuring, Behavioral activation, Relaxation exercises	3	Waitlist or assessment only	PHQ-9, GAD-7, PANAS
Suganuma et al. 2018^69^ (JPN)	Nonclinical	457	70.0	38.07	SABORI	Web-based	Retrieval based	Text-based	CBT only	ABC (Activation, Belief, and Consequence) technique, Relaxation exercises	4	Waitlist or assessment only	WHO-5-J, K10, BADS
Suharwardy et al. 2023^70^ (US)	Nonclinical	152	100.0	34.00	Woebot	Standalone app	Retrieval based	Text-based	Integrative	Psychoeducation, Mood tracking, Cognitive restructuring, Behavioral Activation, Mindfulness	6	Active control	PHQ-9, GAD-7

*CA* conversational agent, *CBT* cognitive behavioral therapy, *BDI-2* Beck Depression Inventory II, *QIDS-SR* Quick Inventory of Depressive Symptoms-Self-Report, *DAS-SF* Dysfunctional Attitude Scale-Short Form, *EQ-5D-5L* EuroQol-5 Dimension-5 Level, *PHQ-8* Patient Health Questionnaire-8, *PHQ-9* Patient Health Questionnaire-9, *GAD-7* Generalized Anxiety Disorder scale, *PSS* Perceived Stress Scale, *PSS-10* Perceived Stress Scale-10, *PANAS* Positive and Negative Affect Schedule, *MFHW* Marburger Screening for Habitual Well-being, *IBS-QOL* Irritable Bowel Syndrome-Quality of Life, *DASS* Depression, Anxiety, and Stress Scales, *SAS* Self-rating Anxiety Scale, *CESD-R* Center for Epidemiologic Studies Depression Scale Revised, *PSWQ* Penn State Worry Questionnaire, *STAI* State-Trait Anxiety Inventory, *SWLS* Satisfaction With Life Scale, *R-UCLA* Revised UCLA Loneliness Scale, *UCLA* UCLA Loneliness Scale, *RS-14* 14-item Resilience Scale, *FS* Flourishing Scale, *HADS* Hospital Anxiety and Depression Scale, *WHO-5-J* World Health Organization-Five Well-Being Index, *K10* Kessler 10, *BADS* Behavioral Activation for Depression Scale, *PHQ-ADS* Patient Health Questionnaire Anxiety and Depression Scale, *HMSE-G-SF* German short form of the Headache Management Self-Efficacy Scale.

### Risk of bias of the included studies

**Fig. 2:**
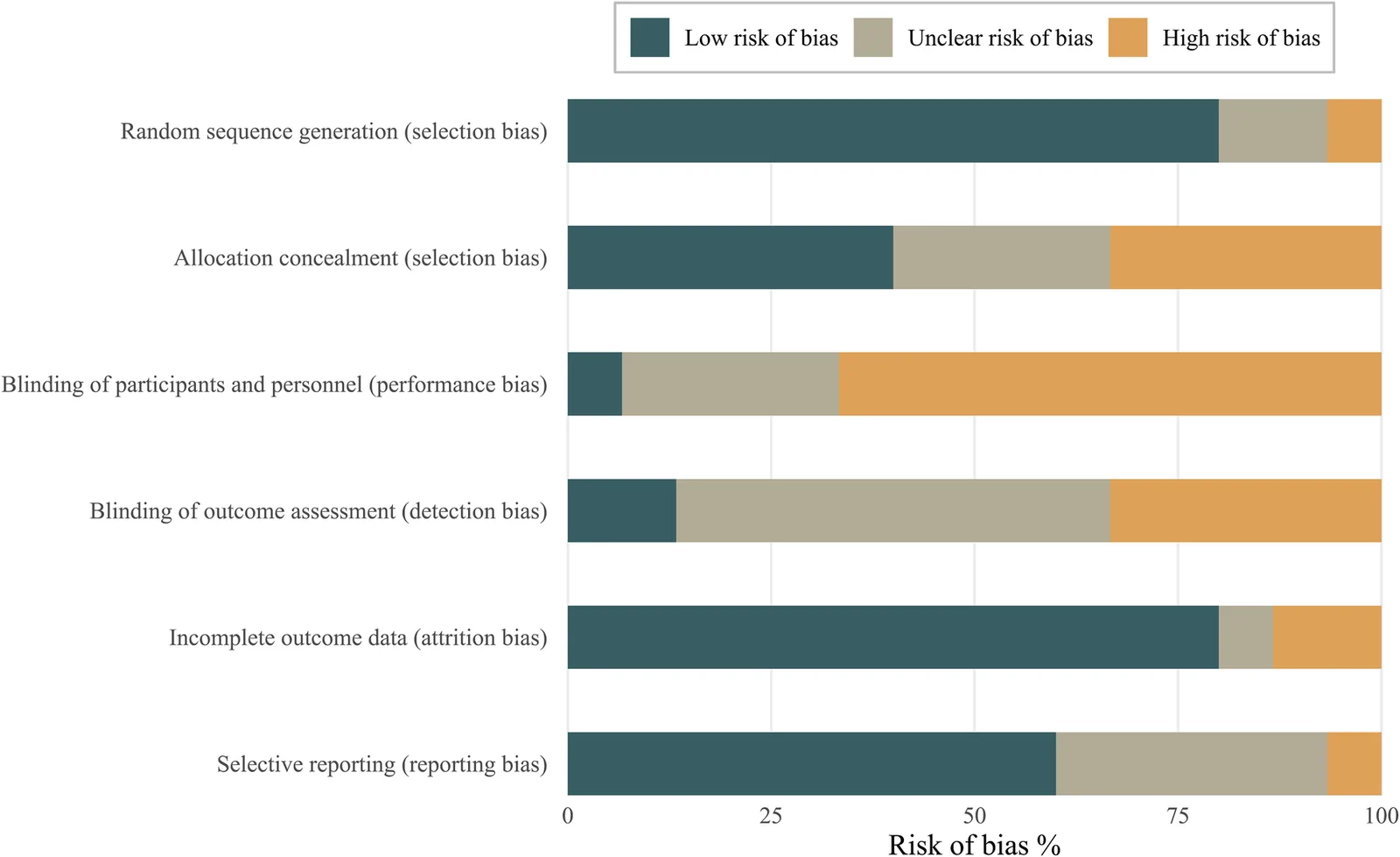
Risk of bias. Authors’ judgments about each risk of bias item were presented as percentages across all included studies. This figure was generated by R (version 4.5.1) https://www.R-project.org/^71^.

Overall, most of the included studies could not be classified as optimal in quality. Only one study met all six of the quality criteria considered^31^. Five studies met four criteria. 1 study met three criteria, while 8 studies met fewer than three. It is important to note that for some criteria, a substantial proportion of the included studies did not provide sufficient information to determine whether these criteria were met. Specifically, 4 out of 15 studies lacked information on blinding of participants or personnel, 8 out of 15 on blinding of outcome assessment, and 5 out of 15 on selective reporting. Additionally, 10 of the 15 studies were found to have a high risk of bias concerning the blinding of participants and personnel. Figure 2 presents the percentage of studies with a low, unclear (i.e., insufficient information), and high risk of bias for each of the quality criteria.

### Depression

**Fig. 3:**
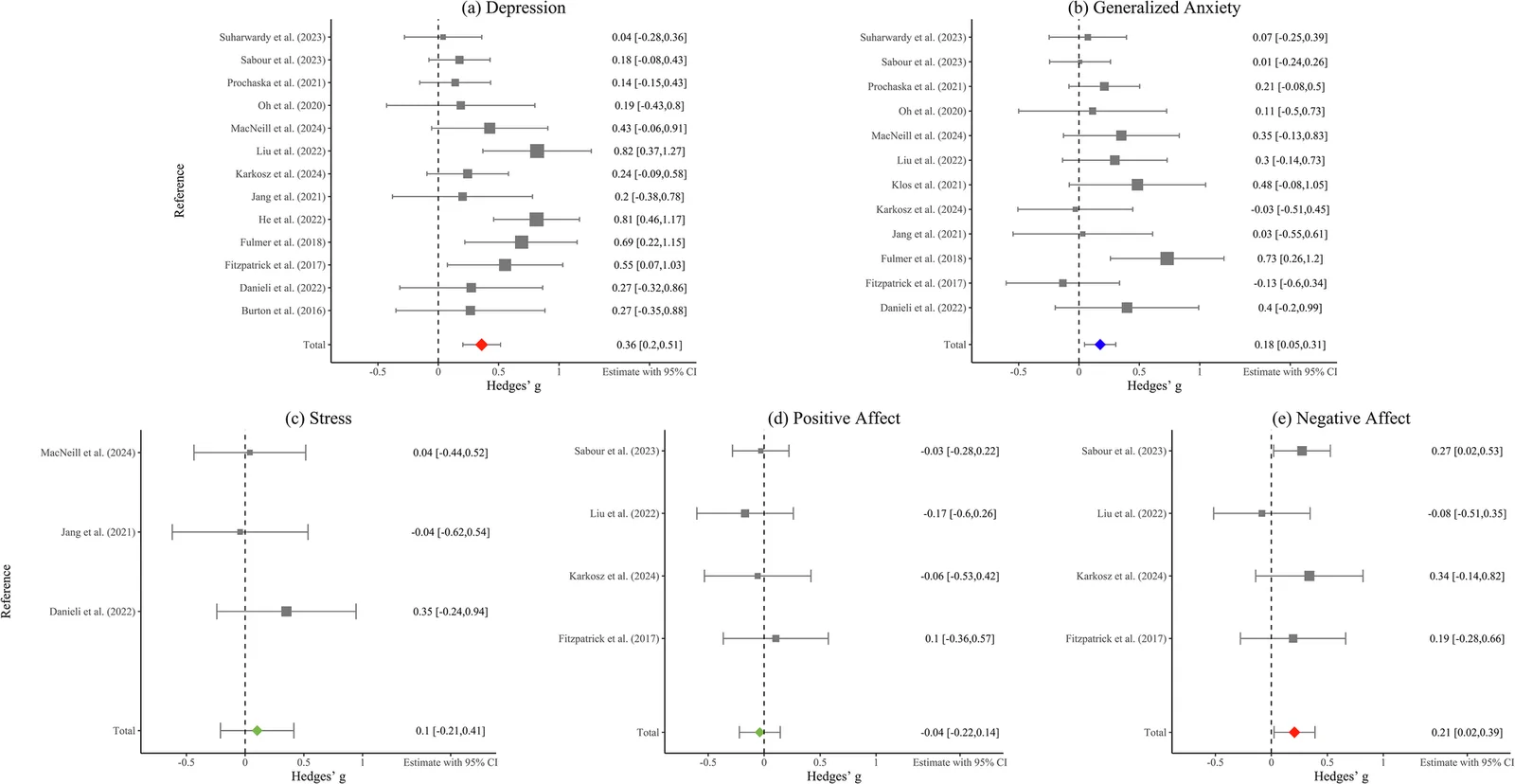
Forest plots for the five outcomes. **a** Depression. **b** Generalized anxiety. **c** Stress. **d** Positive affect. **e** Negative affect. Gray dots indicate the effect size of each study; the red dots indicate the significant total effect size; green dots indicate the non-significant total effect size; blue dots indicate the total effect was not significant when publication bias was controlled. The size of the dots and diamonds indicates the size of effect size; the error bars represent the 95% confidence interval. These plots were generated by R (version 4.5.1) https://www.R-project.org/^71^.

CBT-based CAs demonstrated a small to medium effect on depression symptoms at post-test (*g* = 0.36, 95% CI [0.20, 0.51], *N* = 13, *z* = 4.52, *p* < 0.001) (see Fig. 3a). There was significant heterogeneity among the studies [*Q*(12) = 21.94, *p* < 0.05, *I²* = 45.3%]. No outliers were identified, and sensitivity analyses revealed that the results were not driven by any one study.

**Fig. 4:**
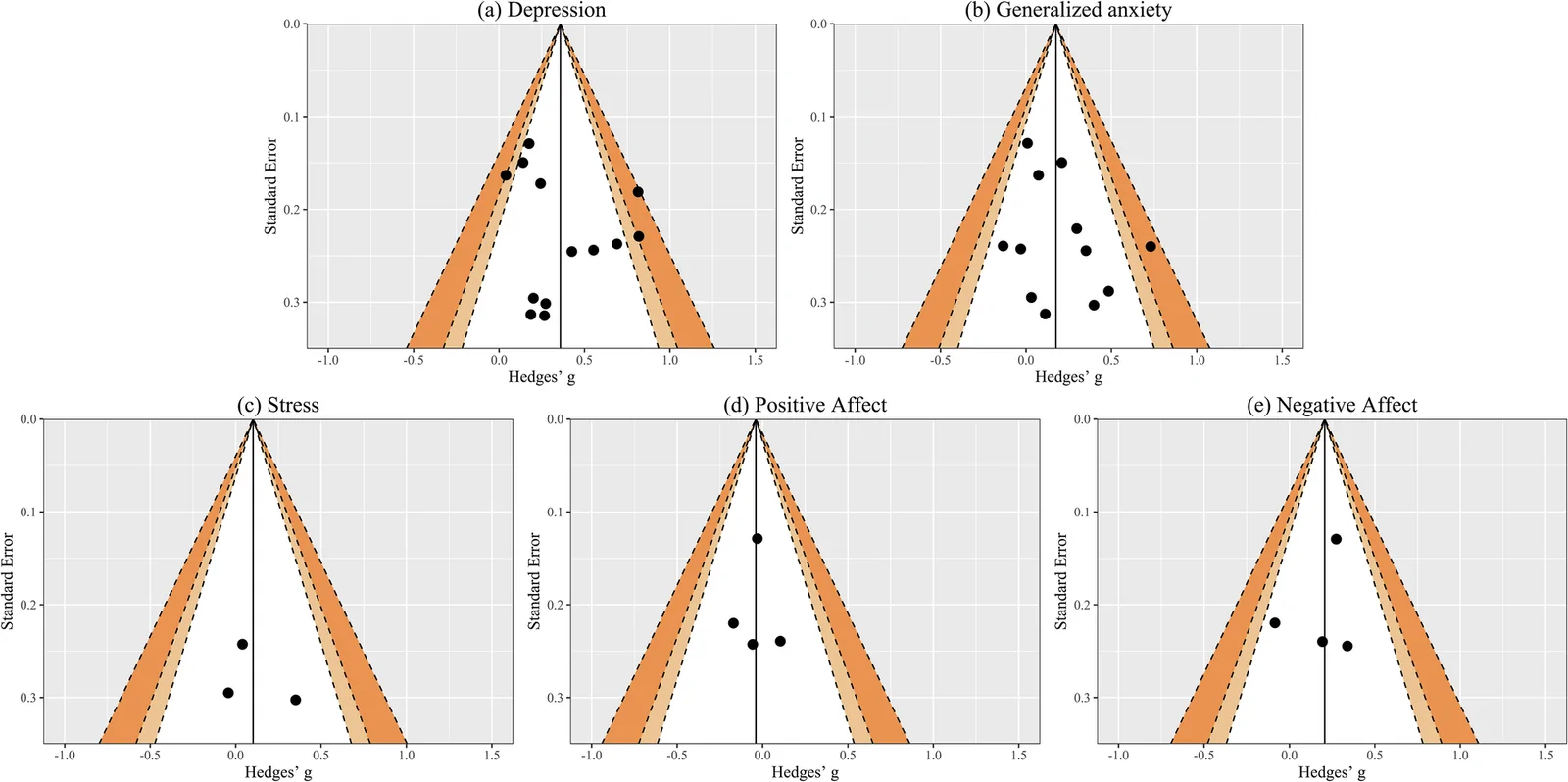
Funnel plots for the five outcomes. **a** Depression. **b** Generalized anxiety. **c** Stress. **d** Positive affect. **e** Negative affect. Each point represents an individual study. The shaded regions indicate contours of statistical significance based on two-tailed *p*-values: white areas correspond to *p* > 0.10, light orange areas to 0.05 < *p* ≤ 0.10, orange areas to 0.01 < *p* ≤ 0.05, and light gray areas outside the main triangle indicate *p* ≤ 0.01. These plots were generated by R (version 4.5.1) https://www.R-project.org/^71^.

Duval and Tweedie’s trim-and-fill procedure did not indicate any evidence of publication bias. Egger’s test revealed similar findings (*b*_*0*_ = 1.48, SE = 1.28, *t* = 1.15, one-tailed *p* = 0.14). The funnel plot is presented in Fig. 4.

**Fig. 5:**
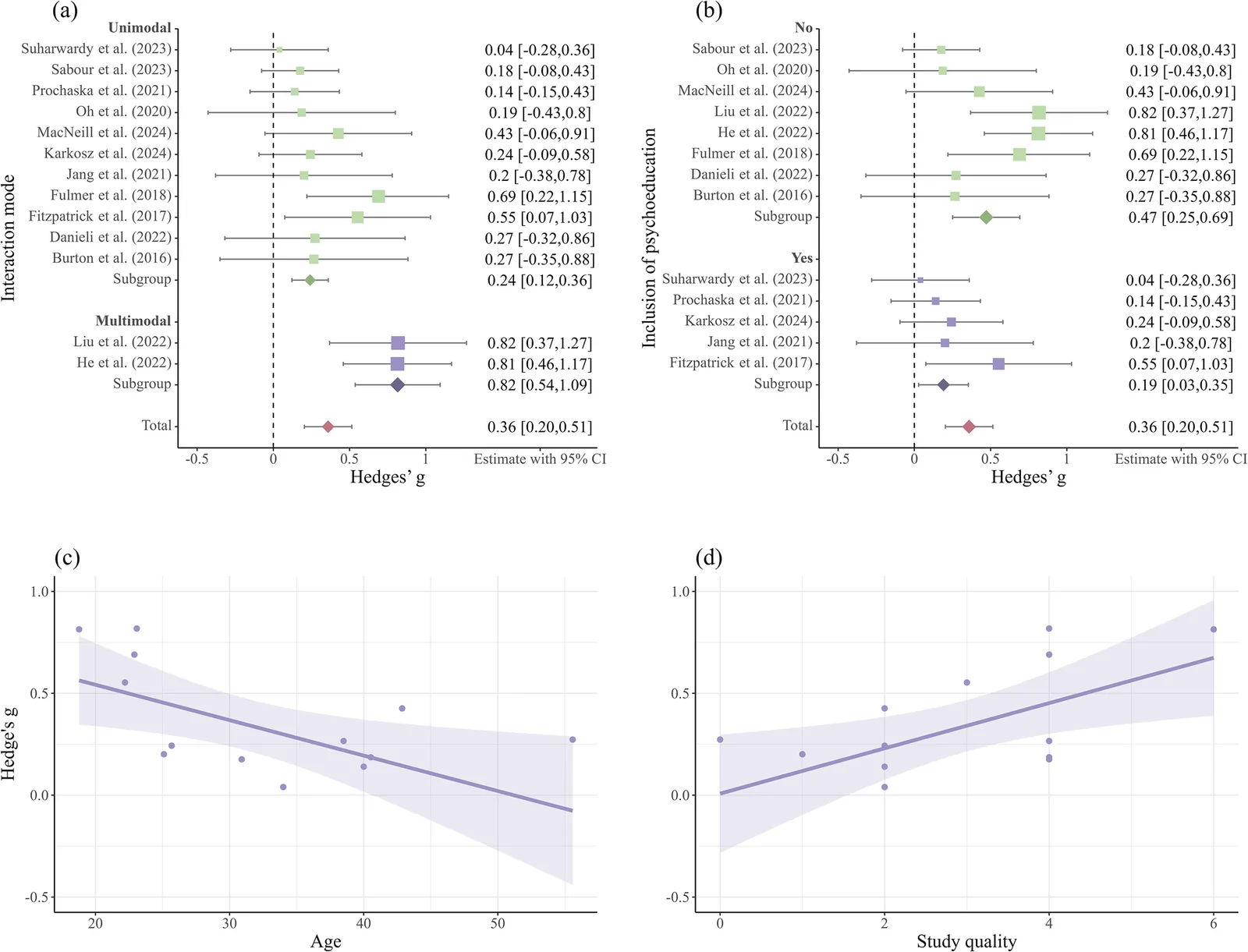
Subgroup analyses and meta-regression addressing depression. **a** Subgroup analysis based on interaction mode of CAs. **b** Subgroup analysis based on inclusion of psychoeducation. **c** Meta-regression with mean participant age as a moderator. **d** Meta-regression with study quality as a moderator. These plots were generated by R (version 4.5.1) https://www.R-project.org/^71^.

Subgroup analyses revealed that interaction mode significantly moderated the post-test ESs (*Q*(1) = 13.84, *p* < 0.001). Larger effect sizes were found in studies that used multimodal CAs (*g* = 0.82, 95% CI = [0.54, 1.09], *Q* = 0.00) (see Table 2 and Fig. 5), and the heterogeneity of the two subgroups both reduce to non-significance (*p* > 0.10, *I²* = 0.0%). Additionally, subgroup analyses regarding CBT skills included in CAs revealed that, the inclusion of psychoeducation significantly moderated the post-test ESs (*Q*(1) = 4.03, *p* < 0.05). Larger effect sizes were found in studies of CAs without psychoeducational contents (*g* = 0.47, 95% CI = [0.25, 0.69], *Q* = 13.56) (see Table 2 and Fig. 5).

**Table 2:** Moderator analyses of the effects of CAs on depressive symptoms

Moderators		*N*	*g*	95% CI	*Qw*	*p*	*Qb*	*p*	*β*	*SE*	*Z*	*p*
Categorical variables												
Sample type	Clinical sample	3	0.32	[−0.01, 0.64]	0.40	0.82	5.25	0.07				
	Nonclinical sample	2	0.12	[−0.08, 0.32]	0.43	0.51						
	Subclinical sample	8	0.47	[0.25, 0.68]	14.64	0.04						
Control group	Active control	4	0.49	[0.05, 0.93]	13.29	0.004	2.47	0.29				
	Information only	5	0.38	[0.17, 0.58]	3.60	0.46						
	Waitlist or assessment only	4	0.20	[0.03, 0.37]	1.10	0.78						
Interaction mode	Text-based/Voice-based	**11**	**0.24**	**[0.12, 0.36]**	**8.10**	**0.62**	**13.84**	**0.000**				
	Multimodal	**2**	**0.82**	**[0.54, 1.09]**	**0.00**	**0.99**						
Delivery platform	Instant messenger platform	5	0.52	[0.22, 0.82]	13.61	0.009	3.20	0.07				
	Standalone app	8	0.22	[0.06, 0.37]	4.14	0.76						
Therapeutic approach	CBT only	10	0.37	[0.19, 0.54]	16.28	0.06	0.00	0.96				
	Integrative	3	0.36	[0.20, 0.52]	5.51	0.06						
Intervention length	0–4 weeks	9	0.40	[0.22, 0.58]	12.01	0.15	0.34	0.56				
	>4 weeks	4	0.36	[0.20, 0.52]	8.33	0.04						
*Specific CBT skills included in CAs*												
Mood tracking	Yes	8	0.37	[0.16, 0.58]	12.88	0.08	0.01	0.92				
	No	5	0.35	[0.09, 0.61]	9.06	0.06						
Cognitive restructuring	Yes	11	0.38	[0.20, 0.55]	21.70	0.02	0.37	0.54				
	No	2	0.24	[−0.18, 0.65]	0.03	0.87						
Behavioral activation	Yes	4	0.19	[−0.01, 0.38]	3.22	0.36	3.05	0.08				
	No	9	0.43	[0.24, 0.63]	15.00	0.06						
Relaxation exercises	Yes	6	0.48	[0.24, 0.73]	6.77	0.24	1.52	0.22				
	No	7	0.29	[0.11, 0.47]	10.74	0.10						
Psychoeducation	Yes	**5**	**0.19**	**[0.03, 0.35]**	**3.27**	**0.51**	**4.03**	**0.045**				
	No	**8**	**0.47**	**[0.25, 0.69]**	**13.56**	**0.06**						
Mindfulness	Yes	7	0.40	[0.15, 0.64]	15.25	0.02	0.32	0.57				
	No	6	0.31	[0.11, 0.50]	6.29	0.28						
Continuous variables												
Mean age		**13**							**−0.02**	**0.007**	**−2.42**	**0.02**
Year		13							−0.03	0.03	−0.82	0.41
%—total Female		13							−0.006	0.006	−1.02	0.31
Quality criteria		**13**							**0.11**	**0.04**	**2.54**	**0.011**

Data are presented as Hedges’ *g* with 95% confidence intervals (CI). Statistical significance is indicated by bold values (*p* < 0.05).

*CA* conversational agent, *CBT* cognitive behavioral therapy, *SE* standard error, *N* number of studies.

As for continuous moderators, the meta-regressions revealed that mean age of participants (*β* = −0.02, *p* < 0.05) and study quality (*β* = 0.11, *p* < 0.05) significantly moderated post-test ESs (see Fig. 5).

### Generalized anxiety

CBT-based CAs had a small but significant effect on generalized anxiety symptoms at post-test compared to the control group (*g* = 0.18, 95% CI [0.05, 0.31], *N* = 12, *z* = 2.66, *p* < 0.01) (see Fig. 3b). No evidence of heterogeneity existed among the studies [*Q*(11) = 12.60, *p* = 0.32, *I²* = 12.7%]. There were no outliers. Sensitivity analyses confirmed that the results were not influenced by any single study.

Duval and Tweedie’s trim-and-fill procedure indicated that two studies were missing on the left side of the mean ES. By assuming a random-effects model, and after missing studies were imputed, the new ES became non-significant (*g* = 0.12, 95% CI = [−0.03, 0.27], *Q* = 21.73, *p* > 0.05). However, Egger’s test failed to find significant publication bias (*b*_*0*_ = 1.28, SE = 1.02, *t* = 1.25, one-tailed *p* = 0.12) (see Fig. 4).

As the adjusted ES was not significant (*p* > 0.05), and the heterogeneity was not significant (*p* = 0.32, *I²* = 12.7%), moderator analyses were not conducted.

### Stress

CBT-based CAs had a non-significant effect on stress at post-test compared to the control group (*g* = 0.10, 95% CI = [−0.21, 0.42], *N* = 3, *z* = 0.65, *p* = 0.52) (see Fig. 3c). Heterogeneity was non-significant among the studies [*Q*(2) = 0.99, *p* = 0.61, *I*^*2*^ = 0.0%]. No outliers were identified, and sensitivity analyses revealed that the results were not driven by any one study.

Duval and Tweedie’s trim-and-fill procedure did not indicate any evidence of publication bias. Egger’s test also revealed a similar finding (*b*_*0*_ = 2.49, SE = 5.18, *t* = 0.48, one-tailed *p* = 0.36) (see Fig. 4).

As the overall ES and heterogeneity were not significant (*p* = 0.52), and the heterogeneity was not significant (*p* = 0.61, *I*^*2*^ = 0.0%), moderator analyses were not conducted.

### Positive affect

CBT-based CAs had a non-significant effect on positive affect at post-test compared to the control group (*g* = −0.04, 95% CI = [−0.22, 0.14], *N* = 4, *z* = −0.42, *p* = 0.68) (see Fig. 3d). There was no evidence of heterogeneity among the studies [*Q*(3) = 0.72, *p* = 0.87, *I*^*2*^ = 0.0%]. No outliers were identified, and sensitivity analyses revealed that the results were not driven by any one study.

Duval and Tweedie’s trim-and-fill procedure did not indicate any evidence of publication bias. Egger’s test also revealed a similar finding (*b*_*0*_ = −0.02, SE = 1.07, *t* = 0.02, one-tailed *p* = 0.49) (see Fig. 4).

As the adjusted ES was not significant (*p* = 0.68), and the heterogeneity was not significant (*p* = 0.87, *I*^*2*^ = 0.0%), moderator analyses were not conducted.

### Negative affect

CBT-based CAs demonstrated a small but significant effect on negative affect at post-test compared to the control group (*g* = 0.21, 95% CI [0.02, 0.39], *N* = 4, *z* = 2.22, *p* = 0.03) (see Fig. 3e). There was no significant heterogeneity among the studies [*Q*(3) = 2.32, *p* = 0.51, *I²* = 0.0%]. No outliers were identified, and sensitivity analyses confirmed that the results were not driven by any individual study.

Duval and Tweedie’s trim-and-fill procedure did not suggest any evidence of publication bias, which is repeated by Egger’s test (*b*_*0*_ = −0.99, SE = 1.77, *t* = 0.56, one-tailed *p* = 0.32) (see Fig. 4).

Given the small overall effect size and the limited number of studies, moderator analyses were not conducted.

## Discussion

This study provides a focused and differentiated synthesis of the existing evidence on CBT-based NLP-enabled AI CAs for various mental health conditions. Potential moderators and publication bias were examined. A total of 15 studies with a combined sample of 1,737 participants were included. Findings indicated that NLP-enabled AI CAs based on CBT can effectively improve depressive symptoms and negative affect, particularly in studies utilizing multimodal CAs and those involving younger adults. The heterogeneities of most outcomes were non-significant, underscoring the consistency and reliability of these findings.

The results indicated that CBT-based NLP-enabled AI CAs had a small to moderate effect on depressive symptoms and a small effect on negative affect. These findings partially align with existing literature, underscoring the potential of NLP-enabled AI CAs to effectively address depression and mitigate negative emotional states^20,28^. The effect size for depressive symptoms in this study was small to moderate, compared to the small effects reported in recent reviews that also included rule-based CAs and CAs based on various theoretical orientations^20,28^. This suggests that CAs enhanced by advanced AI and machine learning technologies may be more effective than their rule-based counterparts in alleviating depressive symptoms.

Compared with depression, NLP-enabled AI CAs based on CBT demonstrate lower efficacy in addressing generalized anxiety, stress, and positive affect. The effect on anxiety became non-significant after adjusting for publication bias. This finding is consistent with two previous meta-analyses that also focused solely on NLP-enabled AI CAs for anxiety^24,27^, while two other meta-analyses that included rule-based CAs found small but significant effects on anxiety^20,28^. Additionally, the non-significant effect on stress differs from the findings of He et al., who reported a small but significant effect on stress. Although CBT is an evidence-based approach for treating anxiety and stress symptoms^32^, it often involves behavioral strategies that directly target fear and avoidance processes, such as exposure-based techniques. However, our structured coding of CBT components in the present review revealed that only one included study^30^ explicitly reported the use of exposure training. The majority of CAs primarily delivered techniques more commonly applied in depression-focused CBT, such as cognitive restructuring and behavioral activation, as also mentioned in another review^33^. Consequently, NLP-enabled AI CAs may need to be integrated with exposure therapy to achieve maximum efficacy in reducing anxiety and stress symptoms.

These mixed outcomes suggest that while NLP-enabled AI CAs based on CBT are particularly effective for certain conditions, such as depression, their impact on anxiety, stress, and positive affect may be limited. To note, as the analyses of the effect on stress, positive affect, and negative affect outcomes were based on a limited number of studies, the robustness and generalizability of the current findings should be interpreted with caution. Similarly, the observed attenuation in significance after publication bias adjustment highlights the need for more robust and consistent evidence before drawing firm conclusions regarding the effectiveness of CAs for generalized anxiety. Additionally, the generally small effect sizes, particularly for outcomes such as negative affect, may limit their clinical significance. Although statistically significant, these small effects may not represent meaningful improvements in clinical practice, especially when the clinical relevance is considered in real-world settings.

Consistent with previous research^25^, subgroup analyses provided preliminary evidence that multimodal CAs—those supporting interactions based on both text, voice and sometimes images—demonstrated superior efficacy in reducing depressive symptoms compared to single-modality CAs. This enhanced efficacy may be attributed to the increased social presence and deeper personalization offered by multimodal CAs, which foster a more human-like experience and amplify therapeutic effects^34,35^. These features of multimodal CAs may be particularly beneficial for depressive symptoms, which are often closely linked to social withdrawal and interpersonal disconnection^36^. By contrast, outcomes such as anxiety, stress, and positive affect may be less sensitive to interaction-mode effects, potentially because these constructs are less directly influenced by perceived social presence. This may explain why significant heterogeneity was observed for depression but not for these other outcomes. These findings suggest that multimodal approaches could potentially represent best practices for optimizing the effectiveness of NLP-enabled AI CAs on depression. However, these findings are based on a limited number of studies, and are likely confounded by design and implementation factors, meaning definitive conclusions cannot yet be drawn. The current results are therefore primarily preliminary and hypothesis-generating. Notably, there was no evidence of heterogeneity for depression within either subgroup (multimodal vs. unimodal), which helps to explain the moderate heterogeneity observed in the overall sample. Taken together, these patterns of heterogeneity also provide some additional support for the methodological decision to focus on CBT-based NLP-enabled AI CAs in the present review, as restricting analyses to a single theoretical orientation may help reduce conceptual heterogeneity and mitigate the “apples and oranges” problem commonly noted in meta-analyses^37^.

Interestingly, subgroup analyses examining specific CBT components suggested that the inclusion of psychoeducational content was associated with smaller post-test effect sizes, whereas CAs without explicit psychoeducation demonstrated comparatively larger effects. One possible explanation is that, the absence of psychoeducation may reflect a more streamlined and focused intervention structure. Simpler and more narrowly targeted CA designs may facilitate more consistent implementation of core CBT mechanisms, enhance usability, and reduce extraneous cognitive load^38^. In contrast, extensive psychoeducation may dilute the delivery of more active, change-oriented CBT techniques (e.g., guided cognitive restructuring), thereby limiting opportunities for experiential learning and practicing. Importantly, this pattern does not imply that psychoeducation is inherently ineffective. Rather, it underscores that the impact of specific CBT components may depend on how they are operationalized and integrated within CAs. Under the broad umbrella of CBT, the effective integration of multiple intervention components—rather than their mere accumulation—appears crucial, as incompatible or poorly coordinated elements may dilute core change mechanisms and attenuate treatment effects^39^. Given the limited number of studies, these findings are preliminary, and more robust evidence is needed to draw firm conclusions. Future research with more detailed reporting of intervention content is needed to clarify how different CBT components interact with CA design features to influence treatment outcomes.

The meta-regression analysis identified age as a significant moderator, with younger adults benefiting more from CBT-based NLP-enabled AI CAs compared to older adults. This trend contrasts with previous findings, which suggested that NLP-enabled AI CAs were more effective for middle-aged and older adults than for adolescents and young adults^20^. However, this discrepancy may be attributable to the broader scope of the study by He et al., which included CAs grounded in diverse theoretical orientations. Our findings indicate that NLP-enabled AI CAs specifically grounded in CBT are more effective for younger adults. One possible explanation is that younger individuals, who already use the internet extensively as a source of health information^40^, are generally more attracted to and receptive of digital mental health interventions^41^. As a result, they may be more willing and able to engage with and apply cognitive-behavioral strategies delivered through digital platforms^42^. These findings highlight the importance of considering age disparity in digital mental health interventions and tailoring these interventions to meet age-specific needs to optimize therapeutic outcomes.

To note, meta-regression revealed a significant relationship between study quality and effect size, with higher-quality studies reporting larger effects. This finding is particularly important as it suggests that current estimates in the literature may be underestimating the true efficacy of CBT-based NLP-enabled AI CAs. Lower-quality studies, which often suffer from methodological limitations such as small sample sizes, unclear protocols, and potential biases, may present less reliable estimates of the intervention’s effectiveness. As such, the true therapeutic potential of these interventions may be more promising than previously thought. This underscores the need for future research to prioritize rigorous study design and high methodological standards to provide more accurate and robust evidence on the efficacy of CBT-based NLP-enabled AI CAs. Ensuring high-quality research will not only improve our understanding of the true impact of these tools but also guide their refinement and real-world implementation.

The findings of this meta-analysis emphasize the substantial clinical potential of NLP-enabled AI CAs based on CBT as scalable and effective interventions for specific mental health conditions, particularly depression. These digital tools address several limitations associated with traditional therapy by offering personalized and adaptive therapeutic experiences tailored to the unique needs of individual patients. Notably, subgroup analyses indicated that younger users derived greater benefits from these interventions, suggesting important implications for targeted implementation. Given that many common mental health disorders, including depression and anxiety, typically emerge during adolescence and early adulthood^43,44^, this age-related effect underscores the potential role of CBT-based NLP-enabled AI CAs in early intervention and prevention. Younger individuals may be especially responsive to AI-delivered CBT due to higher digital literacy and greater familiarity with conversational technologies^42^, highlighting the value of these tools for scalable early-stage mental health care. NLP-enabled AI CAs further enhance CBT’s accessibility by providing flexible and easily accessible support, allowing users to engage with therapeutic content at their own pace and convenience. This flexibility is particularly beneficial for underserved populations who face barriers such as geographical isolation, financial constraints, or social stigma. By lowering these access barriers, CBT-based NLP-enabled AI CAs may lead to increased adherence and engagement, which are critical factors in the success of any therapeutic intervention. Additionally, these platforms can incorporate multimedia elements such as interactive exercises, videos, and real-time feedback, which may further enhance user engagement and facilitate a deeper understanding and application of core CBT principles. Furthermore, NLP-enabled AI CAs may enable language-based assessment^45^ through users’ ongoing naturalistic interactions, allowing for continuous, real-time monitoring of symptom-related expressions, cognitive patterns, and behavioral signals embedded in conversational data. These language-based assessments can facilitate the identification of clinically meaningful patterns over time and may inform more targeted and adaptive CBT interventions. From a methodological perspective, the integration of AI into CBT interventions also provides valuable insights into refining CBT techniques. NLP-enabled AI CAs can collect extensive data on user interactions and outcomes, contributing to a richer evidence base for CBT applications. This data can inform future research and development of CBT methodologies, potentially leading to innovations in how CBT is delivered and optimized for different populations.

However, it is important to note that most of the studies included in this review primarily rely on retrieval-based systems rather than newer generative AI systems. Therefore, the current evidence base primarily reflects the efficacy of retrieval-based AI systems, which are different in their underlying technology and clinical maturity compared to generative models. While the findings of this meta-analysis suggest the potential effectiveness of CBT-based NLP-enabled AI conversational agents, these conclusions should be interpreted with caution and cannot be directly generalized to generative AI systems at this time. Current evidence indicates that generative AI-based counseling systems remain at an early stage of development, characterized by rapid technical experimentation but limited empirical validation. These systems may face significant challenges in maintaining contextual understanding, leading to oversimplified or contextually irrelevant interventions. In addition, generative AI models may present additional ethical risks, including cultural insensitivity and difficulty in managing crisis situations. For instance, generative AI has been found to underestimate suicide risk and may fail to provide timely and appropriate responses in high-risk scenarios^46^. Progress in this area will require standardized evaluation frameworks that combine NLP-based and psychometric measures, transparent reporting of model development and validation practices, and collaboration among clinicians, data scientists, and ethicists to ensure safe, equitable, and clinically accountable deployment^47^.

Several limitations of this study should be acknowledged. First, by including only English-language publications, we may have overlooked relevant studies in other languages, potentially limiting the generalizability of our findings. Second, several outcome domains, particularly stress, positive affect, and negative affect, were informed by a relatively small number of studies. Such analyses should be regarded as exploratory and interpreted with appropriate caution. Similarly, the limited number of studies reporting follow-up effects, coupled with substantial variation in follow-up durations, precluded a thorough examination of the long-term effects of these interventions. As additional trials emerge, future meta-analyses will be better positioned to provide more stable and definitive estimates for these outcomes. Third, this systematic review and meta-analysis was not pre-registered. Although we sought to enhance methodological transparency by explicitly defining the research question, PICO components, eligibility criteria, and analytic strategies prior to data extraction, and by reporting all study selection, data extraction, and analysis procedures in detail, the absence of formal pre-registration remains an important limitation. Specifically, without pre-registration, there is a greater possibility that conscious or unconscious bias may have influenced outcome selection, subgroup analyses, meta-regressions, or other analytic decisions. Therefore, the findings should be interpreted with appropriate caution. Fourth, some subgroup and moderator analyses had limited statistical power due to the small number of studies available. For example, the observed superior efficacy of multimodal CAs for depression is based on only two studies. Although the result reached statistical significance, it should be interpreted with caution and considered a preliminary, hypothesis-generating finding. Future research with larger sample sizes and more studies is needed to confirm and extend these observations. In addition, the present study relied exclusively on quantitative evidence, which limits insight into users’ lived experiences, engagement processes, and ethical perceptions of CBT-based NLP-enabled AI CAs. Future research would benefit from integrating qualitative and mixed-method approaches to more deeply examine therapeutic mechanisms, personalization dynamics, and ethical considerations, in line with emerging methodological standards in mental health research^48^. Finally, due to the small number of studies utilizing generative AIs, we were unable to explore the differential effects of retrieval-based versus generative AIs. Future research could benefit from distinguishing the impacts of these different AI forms.

In conclusion, this meta-analysis provides reliable evidence supporting the efficacy of CBT-based NLP-enabled AI CAs in reducing symptoms of depression and negative affect. While NLP-enabled AI CAs are not intended to replace professional mental health services, our findings suggest their potential as an accessible and effective tool for addressing the growing treatment gap. However, the non-significant effects observed for generalized anxiety, stress, and positive affect after correcting for publication bias indicate that these interventions may be more effective for certain mental health outcomes than others. Future research should aim to refine these NLP-enabled interventions based on frameworks like CBT or other structured approaches such as Acceptance and Commitment Therapy (ACT) to better meet the needs of specific populations, explore their long-term benefits, and address the methodological limitations identified in the current literature.

## Methods

### Literature search

This study was not pre-registered. The current review was guided by PRISMA (Preferred Reporting Items for Systematic Reviews and Meta-Analyses) guidelines (see Supplementary Table 1 for a PRISMA checklist)^29^. To identify studies that examined the effectiveness of NLP-enabled AI CAs based on CBT for adult mental health problems, a systematic search was conducted by two authors (LYN and SXH) independently in PubMed, PsycInfo, Embase, Cochrane library and Web of Science, dated from the establishment of the database to the 1st February 2025. For PubMed, terms related to artificial intelligence CAs (robot OR social bot OR dialogue system OR conversational agent OR conversational bot OR conversational system OR conversational interface OR chatbot OR chat bot OR chatterbot OR chatter bot OR chat-bot OR smartbot OR smart bot OR smart-bot OR virtual coach OR virtual agent OR embodied agent OR relational agent OR avatar OR virtual character OR animated character OR virtual human OR virtual assistant OR digital assistant OR counseling agent) were combined with text words related to mental health outcomes (mental illness OR mental disorder* OR affective disorder OR psychotic disorder OR post-traumatic stress disorder OR PTSD OR distress OR depress OR anxiety OR bipolar OR schizophrenia OR psychosis OR mental health OR mental wellness OR wellbeing OR well-being OR SWB OR happiness OR happy OR positive affect OR negative affect OR positive emotion OR negative emotion OR mood OR life satisfaction OR healthy relationship OR resilience OR self-efficacy). This strategy was then adapted for the other databases (detailed search strategies see supplementary materials). Filters were not utilized to avoid missing any studies that might have presented themselves as experimental studies instead of as randomized controlled trials. The reference lists within the included original studies and previous reviews were manually searched to identify any further eligible studies.

### Inclusion and exclusion criteria

Studies were identified using the following inclusion criteria based on the PICOS framework: (1) Population: adult participants using NLP-enabled AI CAs for their mental health or somatic symptoms were eligible. We defined the clinical population as patients with a formal diagnosis of either physical or mental health conditions. The subclinical population includes individuals who were either screened for or self-identified as having symptoms of mental disorders, such as depression and anxiety, during the study. The nonclinical population consists of participants without self-identified or screened mental health symptoms, or any diagnosed health conditions; (2) Intervention: we included studies employing NLP-enabled AI CAs described by the original authors as being based on CBT or integrative approaches in which CBT represented the primary therapeutic framework and core intervention component, characterized by a two-way interaction between the user and the CA. Integrative interventions were included only when CBT techniques (e.g., cognitive restructuring, behavioral activation, or related CBT-oriented strategies) constituted the dominant therapeutic basis of the intervention, rather than serving as secondary or peripheral elements. Following Li et al., NLP-enabled AI CAs are defined as software agents or bots that utilize NLP, machine learning, or other advanced AI models and techniques to simulate human-like conversations. Unlike rule-based systems that rely on predefined rules to generate responses, these agents are capable of understanding user intent, analyzing context, and generating or retrieving appropriate responses based on the user’s input and the broader context of the conversation; (3) Comparator: we included studies with any comparison, encompassing waitlist or assessment only controls, information-only controls, and active controls (i.e., treatment as usual, therapist-led interventions, or other treatments that did not incorporate CBT-based CAs). (4) Outcome: eligible studies were those that reported at least one mental health outcome and provided the necessary outcome data to calculate effect sizes. (5) Study: only randomized controlled trials (RCTs) were included. Studies conducted on rule-based CAs, commentary articles, review articles, conference abstracts, and non-English articles were excluded. All citations identified through the search were independently reviewed by two authors (WWZ and YK) to ensure reliability. Full texts of all articles that potentially met the inclusion criteria were obtained for independent assessment to determine their eligibility for the review.

### Data extraction and quality assessment

The following characteristics of each study were extracted: authors, year of publication, sample type, sample size, percentage of female participants, mean age, CA name, delivery platform, response generation approach, interaction mode, and therapeutic orientation, intervention length, CBT skills included, type of control group and outcome measures.

The methodological quality of the included studies was assessed using the six criteria from the Cochrane Collaboration’s “Risk of Bias” assessment tool^49^: (1) adequate generation of random allocation sequence, (2) concealment of allocation to conditions, (3) prevention of knowledge of the allocated intervention to participants, (4) prevention of knowledge of the allocated intervention to assessors, (5) dealing with incomplete outcome data, (6) selective outcome reporting. Three authors (WWZ, LYN and SXH) independently extracted data and conducted quality assessment. Disagreements were solved mainly by discussion and if they remained unresolved, another author (HYM) was consulted. The results of the risk of bias assessment are presented as a graph depicting the reviewers’ judgments for each domain.

### Meta-analytic procedure

For each comparison between the treatment group receiving the target CA intervention and the control group, means, standard deviations, and sample sizes at post-test were extracted to calculate effect sizes (ESs). Combined ESs were calculated when more than one RCT was available for a specific outcome, and sufficient data were provided for analysis. Due to the small sample sizes in several studies, the ES was adjusted for small sample bias using Hedges’ *g* with 0.20 indicating a small effect, 0.50 a medium effect, and 0.80 or above a large effect^50^. All ESs were coded so that a positive value of Hedges’ *g* indicated greater improvement in the treatment group compared to the control group, while negative values indicated the opposite^51^. If data from both intention-to-treat and completer analyses were available, the intention-to-treat data were extracted and analyzed. Follow-up measures were not included, as only a limited number of studies provided these data, and the duration of follow-up varied across studies.

The Comprehensive Meta-Analysis software version 3.0 and Stata SE version 15.1 was used to calculate the overall ESs. If a study did not report means and standard deviations for symptom outcomes, other available statistics (e.g., Cohen’s *d*, *t* values or *F* values with corresponding sample sizes) were used to calculate the standardized mean difference. When data for ES calculation were insufficient, the study authors were contacted. For studies with multi-arm designs that included multiple experimental or control groups, we combined the means and standard deviations from the different arms to create a single pairwise comparison, as recommended by the Cochrane guidelines for integrating multiple groups from a single study^52^. Additionally, if multiple instruments were used to measure the same outcome category, an average effect size was calculated.

Given the expected considerable heterogeneity among the studies, the mean ESs were calculated using a random-effects model, which assumes that the included studies are drawn from populations that differ systematically^53^. To assess heterogeneity, the *Q* statistic and the *I²* index were calculated^51^. The *Q* statistic with a *p* value more than 0.10 suggests homogeneity, while *I²* index over 50% and 75% indicate substantial and very high heterogeneity, respectively^54^. Outliers were defined as studies whose 95% confidence intervals fell outside the 95% confidence interval of the pooled studies^55^. For sensitivity analysis, the “leave-one-out” method was employed to identify influential studies and assess the robustness of estimates. Subgroup and meta-regression analyses were then conducted with the outliers excluded.

The publication bias was assessed by three different procedures. First, funnel plots were constructed for various outcome categories, and their asymmetry was visually inspected. Second, Duval and Tweedie’s trim-and-fill procedure^56^ was applied using a random-effects model in CMA (Version 3.0), searching for missing studies to the left of the mean for each outcome category. This method provides an estimate of the ES adjusted for publication bias and indicates the number of missing studies needed to correct funnel plot asymmetry. Third, Egger’s test was performed to assess the symmetry of the funnel plot, with a non-significant intercept indicating no significant asymmetry^57^.

In addition, moderator analyses were conducted when there was evidence of heterogeneity (*p* < 0.10 or *I²* > 25%) and the overall ES was significant. For potential categorical moderators, subgroup analyses were performed using a mixed-effects model, where studies within subgroups were pooled using a random-effects model, and differences between subgroups were tested with a fixed-effects model. For continuous variables, unrestricted maximum likelihood meta-regression analyses were employed to assess whether there was a significant relationship between these variables and the ESs, as indicated by a *Z* value and its corresponding *p* value. Several potential moderating variables (i.e., age, gender, intervention length, interaction mode, delivery platform, therapeutic orientation, sample type and control group type) were selected based on a previous meta-analysis^25^. Additionally, the inclusion of specific CBT components (e.g., behavioral activation, mood tracking, and relaxation exercises) was coded as separate moderator variables in subgroup analyses. Each component was examined individually to assess whether the presence or absence of a given CBT skill within the CAs moderated intervention effects on treatment outcomes. All visualization was conducted by R version 4.5.1.

## Supplementary information

npj-supplementary material_20251223

## Data Availability

Data collected and used in this meta-analysis can be requested from the corresponding author.
